# Tacrolimus toxicity management in a pregnant kidney transplant recipient with newly diagnosed human immunodeficiency virus: A case report

**DOI:** 10.1177/2050313X251317913

**Published:** 2025-02-03

**Authors:** Ahmad Matarneh, Ravi chokshi, Sundus Sardar, Dinia Salmeron, James O’brien, Fareeha Khalil, Naman Trivedi, Nasrollah Ghahramani, Vaqar Shah

**Affiliations:** 1 Department of Nephrology, Pennsylvania State Health Milton S. Hershey Medical Center, Hershey, PA, USA; 2 Department of Maternal-Fetal Medicine, Pennsylvania State Health Milton S. Hershey Medical Center, Hershey, PA, USA

**Keywords:** Tacrolimus toxicity, immune suppression management, cytochrome p450 inducer, HAART, HIV

## Abstract

Managing kidney transplant recipients during pregnancy presents significant challenges, particularly in balancing the interactions and safety concerns of immunosuppressive medications such as mycophenolate mofetil and tacrolimus. Pregnancy can affect tacrolimus levels, and its safety profile during pregnancy remains underexplored. When the patient also hasa human immunodeficiency virus, management becomes even more complicated due to potential interactions between antiretrovirals and immunosuppressants. Notably, tacrolimus is highly susceptible to drug-drug interactions. Even minor adjustments in highly active antiretroviral therapy can result in significant fluctuations in tacrolimus levels, potentially leading to subtherapeutic concentrations (increasing the risk of rejection) or supratherapeutic levels with toxicity. Tacrolimus toxicity is often managed by administering cytochrome P450 enzyme inducers, with the choice of agent depending on factors such as the degree of enzyme induction. Agents such as isoniazid or rifampicin are typically considered. In this case report, we described the treatment of tacrolimus toxicity with rifampicin in a pregnant kidney transplant recipient with a newly diagnosed human immunodeficiency virus infection.

## Introduction

Managing kidney transplant recipients is inherently challenging due to the elevated risk of infections and immune-related complications. For those with human immunodeficiency virus (HIV), this risk is even higher, as both the virus and its treatment further weaken the immune system. In pregnant women, the stakes are even greater due to the risk of mother-to-child transmission of HIV, particularly when viral load is uncontrolled.^1^ Therefore, maintaining viral suppression is critical in pregnancy to minimize the risk of transmission.^2^ Kidney transplant patients with HIV face unique challenges because the combination of highly active antiretroviral therapy (HAART) and immunosuppressive drugs can result in complex drug interactions. Tacrolimus, for example, is prone to adverse effects, and its levels can fluctuate significantly depending on the coadministration of certain HAART medications. Some antiretrovirals can increase tacrolimus concentrations, leading to toxicity, while others may accelerate its metabolism, resulting in subtherapeutic levels.^3^ Tacrolimus toxicity, typically seen when plasma concentrations exceed 15 ng/ml, can present as hypertensive crises, acute renal failure, and, in severe cases, central nervous system symptoms.^4^ Managing tacrolimus toxicity during pregnancy involves careful drug selection, particularly when using P450 inducers. Medications such as phenytoin, carbamazepine, and rifampicin have been employed for this purpose, with rifampicin often regarded as the safest option.^5^ This case report explores the management of a pregnant patient with newly diagnosed HIV who developed tacrolimus toxicity and was successfully treated with rifampicin.

## Case presentation

A 27-year-old female with a past medical history of newly diagnosed HIV, chronic hypertension, and end-stage renal disease secondary to primary focal segmental glomerulosclerosis, followed by bilateral nephrectomies, due to ongoing nephrotic range proteinuria, and a deceased donor kidney transplant 9 years ago with a baseline creatinine around 2.5 mg/dl, presented to our hospital as a transfer at 24 weeks of gestation. The patient had been managed for her pregnancy at an outside institution and was transferred due to acute allograft dysfunction and acute worsening of her hypertension, raising concern for transplant rejection versus superimposed preeclampsia. The patient had stopped all her immunosuppressive medications upon discovering her pregnancy, approximately 4 weeks after becoming pregnant. She had been started on intermittent hemodialysis prior to transfer due to uremia and received a packed red blood cell transfusion for severe anemia. Due to concerns about superimposed preeclampsia and the possible need for preterm delivery, she received betamethasone for fetal lung maturation and magnesium sulfate infusion at a reduced dose for maternal seizure prophylaxis (eclampsia) and fetal neuroprotection. Upon arrival at our hospital, she was started on tacrolimus (0.1 mg/kg/day) 3 mg twice a day and azathioprine 50 mg with a goal tacrolimus level of 4–6 ng/ml.

Her anti-hypertensive regimen was up-titrated to control her blood pressure in a safe range (<160/110). Labs on admission were significant for Blood Urea Nitrogen (BUN)18 mg/dl, creatinine 1.95 mg/dl, CrCl 36 ml/min, and estimated glomerular filtration rate (eGFR) 35 ml/min/1.73 m^2^ (post-dialysis), Hb 9.5 g/dl (post-transfusion), and platelet 215K/µL, normal liver function tests, and a tacrolimus level of 5.4 ng/ml. She completed a maternal-fetal medicine (MFM) ultrasound on admission, which showed normal visualized anatomy and a fetus appropriately grown at 623 g (29%). She was admitted to the MFM antepartum service where she received as needed fetal monitoring and serial labs.

Patient’s HIV had been managed on Biktarvy (bictegravir/emtricitabine/tenofovir alafenamide) with a negative HIV viral load and normal CD4 count prior to transfer. Transplant infectious disease was consulted to help manage her HIV and they recommended discontinuing her Biktarvy due to concerns for worsening renal function and concerns for nephrotoxicty with Biktarvy, they started her on Juluca (dolutegravir 50 mg/rilpivirine 25 mg), darunavir 600 mg bid + ritonavir 100 mg bid as pharmacokinetics of rilpivirine might change in pregnancy; darunavir was added, along with ritonavir as a booster. Due to known interaction between protease inhibitors (ritonavir) and tacrolimus, daily tacrolimus levels were recommended and obtained.

The following day she began to experience malaise, chest discomfort, and diffuse body aches of unclear etiology. Cardiac testing returned negative. In the evening, she was noted to have a lower normal range of blood pressure with a minimum being 100/67. All of her anti-hypertensive medication had to be held to avoid hypotension.

Repeat laboratory evaluation noted K 6.5 mmol/l, HCO_3_ 15 mmol/l, BUN 78 mg/dl, Cr 4.83 mg/dl, CrCl 14 ml/min, eGFR 12 ml/min/1.73 m^2^, and a tacrolimus level of 48.6 ng/ml using chemiluminescent microparticle immunoassay. Given concern for tacrolimus toxicity, her tacrolimus, darunavir, and ritonavir were held and she was upgraded to the intensive care unit (ICU) for closer monitoring. She received hemodialysis for a brief time and was later transitioned to continuous renal replacement therapy (CRRT) due to having lower blood pressure. Infectious disease changed her HAART regimen to dolutegravir, lamivudine, and abacavir (discontinued protease inhibitors) while awaiting resolution of her supratherapeutic tacrolimus level.

She remained on continuous fetal monitoring while in the ICU and on CRRT. Her repeat tacrolimus level continued to uptrend and became elevated beyond the range of laboratory assessment (>60 ng/ml).

**Figure 1.**
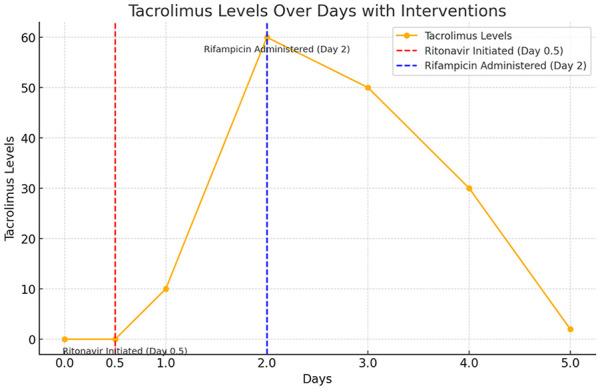
Tacrolimus levels over time. Tacrolimus levels after rifampicin.

After a multidisciplinary meeting between transplant nephrology, MFM, infectious disease, intensive care, and clinical pharmacy, a decision was made to treat the patient with rifampin 600 mg once a day orally for 2 days, to help metabolize the supratherapeutic tacrolimus. The ongoing concern was for severe immunosuppression along with her HIV status and risk of maternal and fetal complications (Figure 1).

During the next 48 h the patient had episodes of acute onset epigastric pain not relieved by intravenous fentanyl, coinciding with acute hypertension requiring nicardipine drip. She underwent laboratory evaluation with normal liver function testing and normal pancreatic enzymes. Multiple rounds of computed tomography imaging with and without contrast did not find an etiology of her pain (negative for liver, bowel, or vascular pathology). The patient appeared to be in acute distress during these episodes, which would then spontaneously resolve. Fetal testing during these episodes was guarded, but there was no evidence of acute decompensation. No obstetric etiology for the pain was evident. It is possible this was secondary to tacrolimus toxicity, and was treated as such with supportive measures and rifampin.

Due to the complex clinical picture, it was unclear if these episodes of hypertension and epigastric pain were related to superimposed preeclampsia. A preeclampsia biomarker test (soluble Fms-like tyrosine kinase-1 (sFLT-1)/placental growth factor (PlGF), Labcorp test 486226) was sent and returned negative (sFLT-1 1856, PlGF 239.5, ratio 8). The clinical utility of this was unclear given that this test has not been validated in the setting of hemodialysis or CRRT.

Ten days after presentation, the patient had a recurrence of acute epigastric pain and hypertension unable to be controlled on nicardipine and labetalol drip. Fetal testing also showed some concerning fetal decelerations and after discussion with the patient, an appropriate consent plan to proceed with emergent cesarean delivery was made. Due to the known low viral load, delivery was not delayed to obtain a zidovudine bolus, however, a 4 g magnesium bolus was given for fetal neuroprotection. She underwent a cesarean section with the delivery of a viable newborn girl. The surgery was complicated by a grade 3 renal capsule injury (renal laceration during closure, greater than 1 cm without injury to the collecting system), and the patient required 4 units of packed red blood cells and 2 units of fresh frozen plasma for resuscitation. Urology team repaired the injury intraoperatively.

Patient recovered in the ICU and did well postoperatively. She had no recurrence of her episodic epigastric pain and she was able to be weaned off nicardipine to an oral regimen. After initially remaining on CRRT, she was able to regain adequate renal function to not require further hemodialysis. Her tacrolimus levels improved and were able to be restarted.

Newborn was noted to be doing well in the neonatal ICU, meeting appropriate goals for gestational age.

## Discussion

Pregnancy after kidney transplantation poses a significant challenge in the setting of altered pharmacokinetics, increased risk of preeclampsia, and complex management of immunosuppressive therapies such as tacrolimus.^6^ Physiological changes associated with pregnancy include a 30%–50% increase in blood volume, altered renal function, and modifications in body composition, which affect drug absorption, distribution, metabolism, and excretion. These changes may hugely influence the levels of tacrolimus and hence require close monitoring with frequent adjustments in dosage to maintain therapeutic efficacy.^7,8^

There are several physiological changes during pregnancy that greatly alter the volume of distribution of tacrolimus. The increased blood volume and cardiac output during pregnancy favor increased distribution, possibly leading to dilution in tacrolimus concentrations. This increased blood volume can result in a lower effective concentration of tacrolimus and thus complicate efforts at maintaining therapeutic levels.^9^ Changes in body composition, such as increased body fat and total body water, may alter the distribution of both lipophilic and hydrophilic drugs. Decreased plasma proteins, such as albumin, increase the free fraction of tacrolimus available to exert a pharmacologic effect and increase the potential for toxicity. These factors contribute to an increased challenge in the management of immune suppression during pregnancy.^10^

The co-management of HIV complicates this further in kidney transplant patients because of the potential for significant drug-drug interactions.^11^ HAART is necessary for the control of HIV, but these medications can interact with immunosuppressive agents to result in subtherapeutic levels or toxicity. Protease inhibitors, a group of drugs including ritonavir, are well-known potent inhibitors of the cytochrome P450 system, primarily CYP3A4, which metabolizes tacrolimus. This inhibition will increase the concentration of tacrolimus, increasing its chances of developing its toxicity. On the other hand, some antiretroviral medications act as inducers of CYP3A4 and can lower levels of tacrolimus, thereby increasing the risk of graft rejection (Table 1).^12,13^ These interactions must be weighed against the need for effective viral suppression, and their balance is another complicating factor in the management of tacrolimus and HIV. Thus, careful monitoring for toxicity is prudent when the medication regimen is changed.^14^

**Table 1.** Major interactions between tacrolimus and HAARTs.

Antiretroviral class	Examples of drugs	Effect on tacrolimus levels	Management recommendations
PIs	Ritonavir, lopinavir, darunavir, atazanavir	Significantly increase levels	Reduce dose and monitor closely
NNRTIs	Efavirenz, nevirapine	Decrease levels	Increase dose and monitor closely
INSTIs	Raltegravir, dolutegravir	Minimal or no significant change	Keep the same and monitor periodically
Pharmacokinetic enhancers (boosters)	Ritonavir (when used as a booster)	Significantly increase levels	Reduce dose and closely monitor
NRTIs	Tenofovir, emtricitabine, lamivudine	Minimal or no significant change	Keep the same and monitor periodically

HAART: highly active antiretroviral therapy; PIs: protease inhibitors; NNRTIs: non-nucleoside reverse transcriptase inhibitors; INSTIs: integrase strand transfer inhibitors; NRTIs: nucleoside reverse transcriptase inhibitors.

Tacrolimus assays are essential in kidney transplant patients to ensure correct dosing. Among these, immunoassays such as Enzyme multiplied immunoassay technique (EMIT) and chemiluminescent microparticle assay (CMIA) are the most widely used because they are fast, automated, and easy for clinical labs to run. However, they sometimes overestimate levels due to cross-reactivity with metabolites. For more precise measurements, liquid chromatography-mass spectrometry (LC-MS/MS) is the “gold standard” because it accurately separates tacrolimus from metabolites, though it takes more time and specialized equipment. High-performance liquid chromatography and radioimmunoassay also measure tacrolimus but are used less frequently. Immunoassays remain the top choice for routine monitoring, while LC-MS/MS is used when results need maximum accuracy.^15^

The symptoms of tacrolimus toxicity are protean and include hypertension, acute kidney injury, and neurological disturbances such as confusion and seizures.^16^ In this patient, the tacrolimus levels increased to toxic levels due to drug interactions with ritonavir, which resulted in worsening renal dysfunction and severe hypertension. Reduction or temporary withdrawal of the drug, along with the use of agents that are cytochrome P450 inducers to increase the metabolism of tacrolimus, usually forms the successful management of tacrolimus toxicity.^17^ Examples are the potent antibiotic rifampin (rifampicin), which, as noted, displays an extremely high induction of tacrolimus breakdown, and the anticonvulsants phenytoin and carbamazepine, which are CYP3A inducers and both effective in their ability to increase the rate of clearance of tacrolimus.^18^ In this instance, rifampicin was chosen for its strong induction of CYP3A4 and its relatively good safety profile during pregnancy. Reduction in tacrolimus levels from over 60 ng/ml to less than 2 ng/ml within 3 days using rifampicin was successful; this approach was thus proven to be effective in cases of severe tacrolimus toxicity.

A multidisciplinary team approach, involving specialists from transplant nephrology, MFM, infectious disease, intensive care, and clinical pharmacy, is necessary to manage the complex drug interactions and physiological changes pregnant patients might experience. Rifampicin offers suitable and safe agents that can be utilized in such situations.

## Conclusion

In this regard, the management of tacrolimus therapy in pregnant patients with HIV represents a complex, multifaceted problem and requires multidisciplinary management of such a complicated scenario. This comprises physiological changes of pregnancy that interact with the interactions of tacrolimus with antiretroviral therapy and requires meticulous monitoring, with individualization of treatment strategies. The successful outcome in this case therefore highlights that even in very complex situations, if handled tactfully and collaboratively, a successful outcome might be anticipated. Rifampicin is an acceptable alternative for the treatment of tacrolimus toxicity in pregnancy.

## References

[1] Simonds RJ . HIV transmission by organ and tissue transplantation. AIDS 1993; 7: S35–S38. 10.1097/00002030-199311002-00008 8161444

[2] Chilaka VN Konje JC . HIV in pregnancy–an update. Eur J Obstet Gynecol Reprod Biol 2021; 256: 484–491. 33246666 10.1016/j.ejogrb.2020.11.034 PMC7659513

[3] Izzedine H Launay-Vacher V Baumelou A , et al. Antiretroviral and immunosuppressive drug-drug interactions: an update. Kidney Int 2004; 66(2): 532–541. 15253704 10.1111/j.1523-1755.2004.00772.x

[4] O’Connor AD Rusyniak DE Mowry J . Acute tacrolimus toxicity in a non-transplant patient. Clin Toxicol 2008; 46(9): 838–840. 10.1080/15563650801898528 18608277

[5] Mrvos R Hodgman M Krenzelok EP . Tacrolimus (FK 506) overdose: a report of five cases. J Toxicol Clin Toxicol 1997; 35(4): 395–399. 9204100 10.3109/15563659709043372

[6] Suarez ML Parker AS Cheungpasitporn W . Pregnancy in kidney transplant recipients. Adv Chronic Kidney Dis 2020; 27(6): 486–498. 33328065 10.1053/j.ackd.2020.06.004

[7] Hebert MF Zheng S Hays K , et al. Interpreting tacrolimus concentrations during pregnancy and postpartum. Transplantation 2013; 95(7): 908–915. 23274970 10.1097/TP.0b013e318278d367 PMC3637974

[8] Le HL Francke MI Andrews LM , et al. Usage of tacrolimus and mycophenolic acid during conception, pregnancy, and lactation, and its implications for therapeutic drug monitoring: a systematic critical review. Therpeutic Drug Monit 2020; 42(4): 518–531. 10.1097/FTD.0000000000000769 32398419

[9] Carlin A Alfirevic Z . Physiological changes of pregnancy and monitoring. Best Pract Res Clin Obstet Gynaecol 2008; 22(5): 801–823. 18760680 10.1016/j.bpobgyn.2008.06.005

[10] Cheung KL Lafayette RA . Renal physiology of pregnancy. Adv Chronic Kidney Dis 2013; 20(3): 209–214. 23928384 10.1053/j.ackd.2013.01.012 PMC4089195

[11] Sorohan BM Ismail G Leca N . Immunosuppression in HIV-positive kidney transplant recipients. Curr Opin Organ Transplant 2023; 28(4): 279–289. 37219235 10.1097/MOT.0000000000001076

[12] Milosh B Bugaighis M Cervia J . Systematic review of the impact of protease-inhibitor-based combination antiretroviral therapy on renal transplant outcomes in recipients living with HIV infection. J Investig Med 2024; 72(7): 776–783. 10.1177/10815589241252595 38666448

[13] Jain AK Venkataramanan R Shapiro R , et al. The interaction between antiretroviral agents and tacrolimus in liver and kidney transplant patients. Liver Transplant 2002; 8(9): 841–845. 10.1053/jlts.2002.34880 12200788

[14] Diana NE Naicker S . The changing landscape of HIV-associated kidney disease. Nature Rev Nephrol 2024; 20(5): 330–346. 38273026 10.1038/s41581-023-00801-1

[15] Murthy JN Davis DL Yatscoff RW , et al. Tacrolimus metabolite cross-reactivity in different tacrolimus assays. Clin Biochem 1998; 31(8): 613–617. 9876892 10.1016/s0009-9120(98)00086-1

[16] Snee I Drobina J Mazer-Amirshahi M . Tacrolimus toxicity due to enzyme inhibition from ritonavir. Am J Emerg Med 2023; 69: 218.e5–218.e7. 10.1016/j.ajem.2023.04.045 PMC10159930 37173153

[17] Kwon EJ Yun GA Park S , et al. Treatment of acute tacrolimus toxicity with phenytoin after Paxlovid (nirmatrelvir/ritonavir) administration in a kidney transplant recipient. Kidney Res Clin Pract 2022; 41(6): 768–770. 36474331 10.23876/j.krcp.22.218 PMC9731781

[18] Van Schalkwyk M Bekker A Decloedt E , et al. Pharmacokinetics and safety of first-line tuberculosis drugs rifampin, isoniazid, ethambutol, and pyrazinamide during pregnancy and postpartum: results from IMPAACT P1026s. Antimicrob Agents Chemother 2023; 67(11): e0073723. 10.1128/aac.00737-23 PMC10648924 37882552

